# Supplementary material for: A Participatory Intervention to Improve the Psychosocial Work Environment and Mental Health in Human Service Organisations. A Mixed Methods Evaluation Study
Source: Int J Environ Res Public Health. 2021 Mar 29;18(7):3546. doi: 10.3390/ijerph18073546 (PMC8037176; doi:10.3390/ijerph18073546)
Supplement: Supplementary file 1 [file ijerph-18-03546-s001.pdf]

Table S1. Description of participants in the interviews and which domains in the Nielsen and Randal framework (Nielsen & Randall, 2013) they were questioned about.

Time<br>Participant characteristic	February 2017	October 2017	May 2018	
**Early childhood and childhood education**				Domains from the Nielsen and Randall model included in the interview guide
Administration manager	x	x	quit	Context, intervention and implementation design and mental models
First line manager 1 (principal)	x	x	quit	ibid
First line manager 2 (principal)	-	-	x	ibid
Team manager administrative unit	x	x	x	ibid
Teacher		x	x	ibid
School counsellor	x	x	x	ibid
Administrative employee	x		x	ibid
Department manager control group	x	retired	–	Context (parallel events within that department)
Total	6	5	5	
**Social services**				
Department manager	x	x	x	Context, intervention and implementation design and mental models
First line manager	x	x	x	ibid
Team leader team 1	x	x	x	
Team leader team 2	x	x	parental leave	
Front-line employee a team 1	x	x	x	
Front-line employee b team 1	x	x	x	
Front-line employee a team 2	x	x	x	
Front-line employee b team 2	x	x	x	
Department manager control group	x	-	x	Context (parallel events within that department)
Total	9	8	8	
Project management and consultants				
Project manager	x	x	x	Context and intervention and implementation design
Consultant 1	x	x	x	ibid
Consultant 2	x	quit		ibid
Total	3	2	3	

Supplementary Material S2. Information on items and Cronbach alpha for all scales.

Psychosocial work environment

The scale score was calculated as the mean of the items, with higher scores indicating a better work environment for the following scales: **Role clarity** (3 items, e.g., "Do you know what your responsibilities are"?), Cronbach's alpha for this scale = 0.80**. Empowering leadership** (3 items, e.g., "Does your immediate superior help you develop your skills"?), Cronbach's alpha for this scale = 0.88. **Social support from manager** (3 items, e.g., "Are your work achievements appreciated by your immediate superior"?), Cronbach's alpha for this scale = 0.87. **Social support from colleagues** (2 items, e.g., "If needed, can you get support and help with your work from your co-workers"?), Cronbach's alpha for this scale = 0.77. **Control of decision** (5 items, e.g., "Can you influence the amount of work assigned to you"?), Cronbach's alpha for this scale = 0.63. **Control of work pacing** (4 items, e.g., "Can you set your own work pace"?), Cronbach's alpha for this scale = 0.69. For the following scales lower scores indicating a better work environment: **Quantitative job demands** (4 items, e.g., "Do you have too much to do"?), Cronbach's alpha for this scale = 0.80. **Decision demands** (3 items, e.g., "Does your work require maximum attention"?), Cronbach's alpha for this scale = 0.68. **Role conflict** (3 items, e.g., "Do you have to do things that you feel should be done differently"?), Cronbach's alpha for this scale = 0.76. The five response alternatives ranged from 1 (very seldom or never) to 5 (very often or always).

Information on **Quality of sleep** was collected from one sub-scale of the Karolinska Sleep Questionnaire (Nordin, Åkerstedt, & Nordin, 2013), which consists of 18 items. The scale comprises the question: "How often have you been troubled by the following in the last 3 months?" with four different symptoms of sleep disturbance following, Cronbach's alpha for this scale = 0.84. The scale score was calculated as the mean of the items (range 1 = never to 6 = always), with higher scores indicating lower quality of sleep.

Covariates

Educational level (Elementary school = 9 years, Upper elementary school > 9 years, University/college). **Occupation within school** (Teacher, Early childhood educator, Recreation leader, Other). **Occupation within social services** (Care assistant, Assistant nurse, Nurse, Cleaner). **Job tenure** (<1, 1–2, 3–5, >5). **Sex** (Female, Male). **Age** (<35, 36–45, >46). **Work-time** (Chosen part-time, Not chosen part-time, Full-time). **Answered before at 24 moths** (Yes, both, Answered one of them, No, none of them, Don’t remember).

Supplementary Material S3. Description of the weighting procedure, comparison between intervention and control groups at different time points, comparison within intervention and control groups at different time points and the way of testing for mass significance.

Comparisons between intervention and non-intervention groups at specific time points

In order to be able to compare if a significant change had been taken place between the intervention and the non-intervention groups at time k, two different weighting procedures were done.

The first one concerns weighting for background variables. Our solution was to first observe the percentages of respondents to response alternatives for five different background variables at time k (age class, type of occupation, working hours, number of years worked at the workplace and education). New variables representing these background variables were created and instead of the actual codes (e.g. 1, 2, 3, 4, 5, 6 and 7) percentages were used, i. e all individuals having responded 1 to a variable were given the same percentage in this new variable. Next, these five created variables were multiplied by each other into a sixth variable, in the following denoted as the background weight variable. Hence, larger values according to this variable indicated that the individual's response pattern was more common compared to others. For each of the nine outcomes, separate conditions were made, i.e. the specific outcome should not be missing. Furthermore, separate frequency tables of the background weight variable revealed the sum was far from the actual number of individuals included depending on the test situation. Obviously, this was due to substantially small values when having multiplied several percentages to each other. Therefore, in order to being able to trust the p-values from t-tests not to be an artifact explained due to small number of degrees of freedom, a ratio was calculated = number of individuals divided by the sum of the background weight variable according to the frequency table. The final weight variable used when doing the t-tests consisted of a multiplication of the ratio and the background weight variable for the specific individual.

The second one concerns weighting for the outcome at k-1, i.e. this weighting was only done when comparisons were made at time point 2 and 3. The ratio became much smaller since the sum of weights to the specific outcome based on time point k-1 was much larger, not having multiplied several percentages to each other.

Moreover, when comparing the intervention and non-intervention groups at time point 3, not only was the difference weighted for the outcome responses at time point 2 but also separately weighted for the outcome responses at time point 1.

Comparisons between time points within intervention and non-intervention groups respectively

Since individuals were not receiving any identification number in the study there was not possible to follow an individual's response over time. As a consequence, it is impossible to know for certain which individual did or did not responded more than once. There exist formulas for doing t-tests when having overlapping samples which we have used in this study. In these formulas the researcher only need to know the number of individuals having responded at both occasions. In the questionnaire at time point k the individual was asked to respond whether having completed the same questionnaire at time point k-1. Although, recall bias might to some extent have occurred there is no reason to believe it should be correlated to the self-reported severity of the outcome variables. Only weighting for background variables was done in these kinds of analyses. Since there were six types of t-tests, i.e. time point 1 vs 2, 1 vs 3, 2 vs 3 among the interventions as well as the non-intervention groups six different frequency tables were run for each background variable including individuals answered only at time point k-1/k-2, only at the time point k or at both occasions. As explained earlier ratios had to be calculated since the sum of the background weight variable was far from equal to the number of individuals depending on the test situation. Again, the final weight variable used when doing the t-tests consisted of a multiplication of the ratio and the background weight variable for the specific individual.

The formula used when the assumption of equal variances holds is:

$$T_{new1} = \frac{\bar{X}_1 - \bar{X}_2}{S_p\sqrt{\frac{1}{n_1}+\frac{1}{n_2}-2r\left(\frac{n_c}{n_1 n_2}\right)}} \quad \text{where } S_p = \sqrt{\frac{(n_1-1)S_1^2+(n_2-1)S_2^2}{(n_1-1)+(n_2-1)}}$$

The test statistic T_{new1} is referenced against the t-distribution with degrees of freedom defined as:

$v_{new1} = (n_c - 1) + \left(\frac{n_a + n_b + n_c - 1}{n_a + n_b + 2n_c}\right)(n_a + n_b)$

n_1= total number of observations in sample at time k

n_2= total number of observations in sample at time k+1

n_a= number of observations exclusive in sample at time k

n_b= number of observations exclusive in sample at time k+1

n_c= number of observations in both samples

r = Pearson's correlation coefficient for the paired observations

However, calculating a correlation was not possible because no individual could be matched between time points. Therefore, several test statistics were calculated assuming nine different correlations, i.e. 1, 0.75, 0.5, 0.25, 0, -0.25, –0.5, –0.75 and –1 respectively. This mode of procedure enabled us to observe the maximum and minimum possible test statistics and as it turned out the conclusions whether to reject or not the hypothesis of no change in mean values between time points were almost always the same.

The formula used when the assumption of equal variances does not hold is:

$$T_{new2} = \frac{\bar{X}_1 - \bar{X}_2}{\sqrt{\frac{S_1^2}{n_1} + \frac{S_2^2}{n_2} - 2r\left(\frac{S_1 S_2 n_c}{n_1 n_2}\right)}}$$

The test statistic T_{new1} is referenced against the t-distribution with degrees of freedom defined as:

$v_{new2} = (n_c - 1) + \left(\frac{\gamma - n_c - 1}{n_a + n_b + 2n_c}\right)(n_a + n_b)$

Where $\gamma = \frac{\left(\frac{S_1^2}{n_1} + \frac{S_2^2}{n_2}\right)^2}{\left(\frac{S_1^2}{n_1}\right)^2 / (n_1 - 1) + \left(\frac{S_2^2}{n_2}\right)^2 / (n_2 - 1)}$

The problem of mass significance was handled by multiplying all the unadjusted p-values by 99 and all adjusted p-values by 132 among home care personnel and staff in school separately. The motivation for choosing 99 is that nine outcomes were studied at three occasions, 11*3 = 33 as well as six specific differences between time points among either intervention or non-intervention groups were done for the same nine outcomes, 11*6 = 66. The motivation for choosing 132 is the additional tests, besides weighting for the background variable, that occur when weighting for the outcome variable at time point k-1, 22 tests, and/or time point k-2, 11 tests, in cases interest lies in comparing intervention and non-intervention groups at specific time points.
